# Potent Anti-Diabetic Effects of MHY908, a Newly Synthesized PPAR α/γ Dual Agonist in db/db Mice

**DOI:** 10.1371/journal.pone.0078815

**Published:** 2013-11-14

**Authors:** Min Hi Park, Ji Young Park, Hye Jin Lee, Dae Hyun Kim, Daeui Park, Hyoung Oh Jeong, Chan Hum Park, Pusoon Chun, Hyung Ryong Moon, Hae Young Chung

**Affiliations:** 1 Molecular Inflammation Research Center for Aging intervention (MRCA), College of Pharmacy, Pusan National University, Busan, Korea; 2 Laboratory of Biochemistry, Pusan National University, Busan, Korea; 3 Laboratory of Medicinal Chemistry, College of Pharmacy, Pusan National University, Busan, Korea; 4 College of Pharmacy, Inje University, Gimhae, Gyeongnam, Korea; National Institute of Nutrition, India

## Abstract

Peroxisome proliferator-activated receptor (PPAR) α/γ dual agonists have been developed to alleviate metabolic disorders and have the potential to be used as therapeutic agents for the treatment of type 2 diabetes. In this study, we investigated the effects of a newly synthesized PPAR α/γ dual agonist, 2-[4-(5-chlorobenzo [*d*] thiazol-2-yl) phenoxy]-2-methylpropanoic acid (MHY908) on type 2 diabetes *in vitro* and *in vivo*. To obtain initial evidence that MHY908 acts as a PPAR α/γ dual agonist, ChIP and reporter gene assays were conducted in AC2F rat liver cells, and to investigate the anti-diabetic effects and molecular mechanisms, eight-week-old, male db/db mice were allowed to eat *ad libitum*, placed on calorie restriction, or administered MHY908 (1 mg or 3 mg/kg/day) mixed in food for 4 weeks. Age-matched male db/m lean mice served as non-diabetic controls. It was found that MHY908 enhanced the binding and transcriptional activity of PPAR α and γ in AC2F cells, and it reduced serum glucose, triglyceride, and insulin levels, however increased adiponectin levels without body weight gain. In addition, MHY908 significantly improved hepatic steatosis by enhancing CPT-1 levels. Remarkably, MHY908 reduced endoplasmic reticulum (ER) stress and c-Jun N-terminal kinase (JNK) activation in the livers of db/db mice, and subsequently reduced insulin resistance. The study shows MHY908 has beneficial effects on type 2 diabetes by simultaneously activating PPAR α/γ and improving ER stress, and suggests that MHY908 could have a potent anti-diabetic effect as a PPAR α/γ dual agonist, and potential for the treatment of type 2 diabetes.

## Introduction

The worldwide prevalence of obesity is steadily growing, and various systems that modulate the balance between energy intake and energy expenditure have been suggested to stave off obesity [1]. Energy homeostasis is regulated by metabolic organs, such as, the liver, adipose tissues, and muscles, more so by liver as it is responsible for energy storage and supply. Alterations in liver function affect whole-body metabolism and energy homeostasis, and importantly underlie the development of metabolic diseases, such as, hyperglycemia, hyperlipidemia, fatty liver, insulin resistance, type 2 diabetes, and metabolic syndrome. Accumulating evidence suggests endoplasmic reticulum (ER) stress plays a substantial role in the pathogenesis of diabetes and contributes to insulin resistance [2], [3], and it has been established that several PPAR α/γ dual agonists enhance insulin sensitivity by inhibiting ER stress [4], [5]. As a result, numerous therapeutics targeting glucose and lipid metabolism have been developed to treat glucose and lipid dysregulation, and its related complications.

PPARs are a group of three homologous transcription factors that belong to the nuclear receptor superfamily, members of which are activated by fatty acids and fatty acid metabolites. The different PPAR subtypes, such as, PPAR α, PPAR γ, and PPAR β/δ, have important physiological functions that are in part determined by their tissue distributions [6]. Recent studies suggest PPAR α activation stimulates lipid consumption by enhancing the expressions of genes that oxidize fatty acids, and that this ameliorates hyperlipidemia [7], [8]. On the other hand, PPAR γ controls lipid mobilization into adipocytes by promoting adipogenesis and regulating the expressions of adipocyte-secreted proteins and adipocytokines, such as, leptin and adiponectin, thereby reducing lipotoxicity [9].

Elevated triglyceride and low high-density lipoprotein cholesterol (HDL-C) levels can be addressed by administering PPAR α agonists, and the insulin-sensitizing effects of these agonists have been demonstrated in a variety of experimental models. Accordingly, PPAR γ agonists are now being used to treat type 2 diabetes [10]. Nevertheless, anti-diabetic drugs are required to treat hyperglycemia and dyslipidemia. In theory, PPAR α/γ dual agonist should be useful for treating major metabolic disorders, such as, hyperglycemia, dyslipidemia and insulin resistance, associated with type 2 diabetes, and for improving complications resulting from aberrant blood glucose and lipid control in metabolic syndrome.

**Figure 1:**
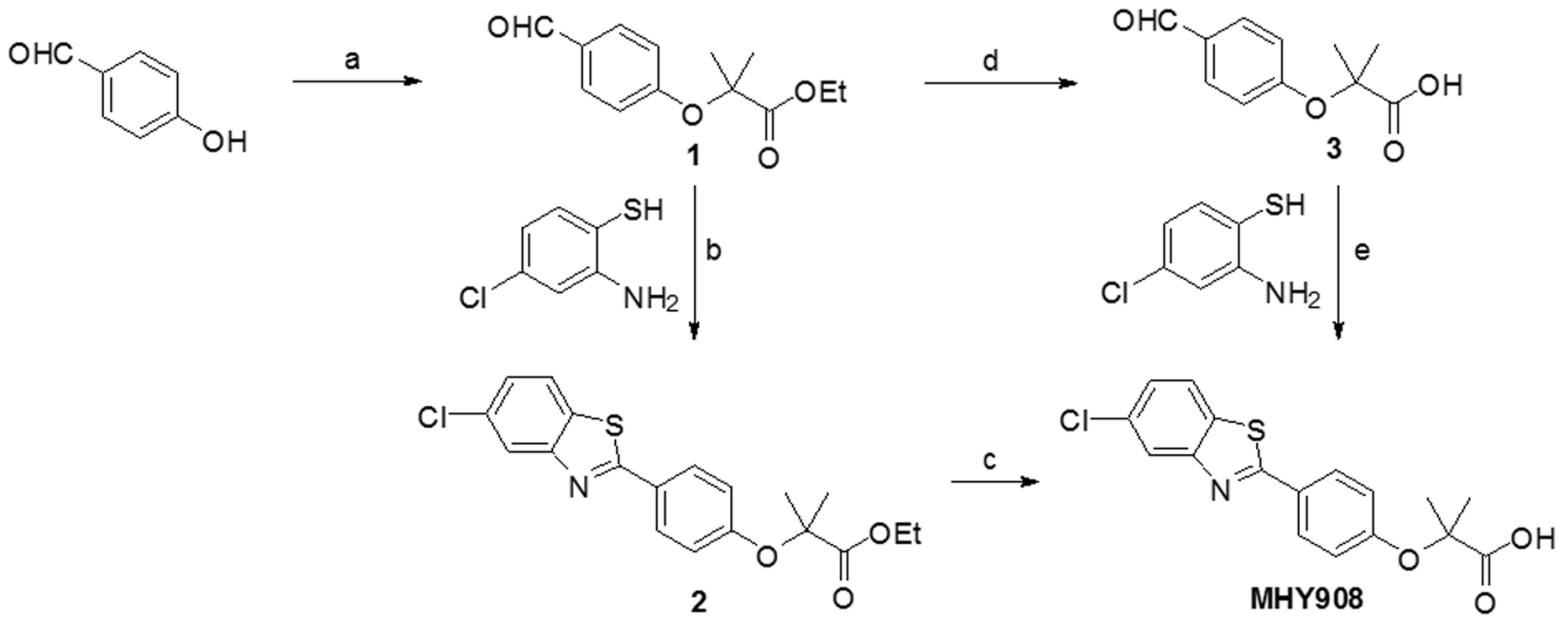
Synthesis of MHY908. Reagents and conditions: (a) Ethyl-bromoisobutyrate, 1N-NaOEt, EtOH, reflux, 14 h, 72%; (b) Na_2_S_2_O_5_, DMF, 80°C, 11 h, 31%; (c) 1N-NaOH, 1,4-dioxane, rt, 17 h, 79%; (d) 1N-NaOH, 1,4-dioxane, rt, 4 h, 99.9%; (e) NaOAc, AcOH, reflux, 1 h, 40%.

A number of PPAR α/γ dual agonists have been recently developed and others are being evaluated for their effects on insulin resistance in animal models and in type 2 diabetes patients [11], [12], [13], [14], [15], [16]. However, many PPAR α/γ dual agonists have failed US FDA clinical trials due to undesirable side effects. Thus, we considered that an agonist that could simultaneously activate PPAR α and γ (Fig. 1) might provide an alternative strategy for the treatment of insulin resistance and dyslipidemia without undesirable side effect. Accordingly, this study was undertaken to identify such a PPAR α/γ dual agonist using a cell culture system and fenofibrate and rosiglitazone (known PPAR α and γ agonists, respectively). Subsequently, we explored the hypothesis that the beneficial effects of MHY908 on type 2 diabetes are due to the inhibition of endoplasmic reticulum (ER) stress and c-Jun N-terminal kinase (JNK) activation in the livers of db/db mice.

## Materials and Methods

### Synthesis and chemical properties of MHY908

1 N sodium ethoxide (127 mL, 127.0 mmol) was added to a stirred solution of 4-hydroxybenzaldehyde (10 g, 81.89 mmol) and ethyl–bromoisobutyrate (18.6 mL, 126.73 mmol) in ethyl alcohol (50 mL) and refluxed for 14 h. After evaporating volatiles, the residue was partitioned between ethyl acetate and water and the organic layer was dried over MgSO_4_, filtered, and evaporated under reduced pressure. The resultant residue was purified by silica gel column chromatography using hexane and ethyl acetate (7:1) as eluent to give compound **1** (13.99 g, 72%): ^1^H NMR (400 MHz, DMSO-*d*_6_) *δ* 9.70 (s, 1 hydrogen, carbohydrate), 7.62 (d, 2 H, *J* = 8.8 Hz, 3′-H, 5′-H), 6.73 (d, 2 H, *J* = 8.8 Hz, 2′-H, 6′-H), 4.05 (q, 2 H, *J* = 6.8 Hz, C*H_2_*CH_3_), 1.50 (s, 6 H, 2×CH_3_), 1.03 (t, 3 H, *J* = 7.2 Hz, CH_2_C*H_3_*); ^13^C NMR (100 MHz, DMSO-*d*_6_) *δ* 190.7, 173.4, 161.0, 131.6, 130.4, 117.8, 79.6, 61.7, 25.4, 14.0; LRMS (ESI) *m/z* 237.2 (M+H)^+^;

A solution of 2-amino-4-chlorobenzenethiol (0.70 g, 4.38 mmol) and compound 1 (1.036 g, 4.38 mmol) in dimethylformamide (10 mL) was then heated at 80°C in the presence of Sodium metabisulfite (834 mg, 4.38 mmol) for 11 h. After evaporating volatiles, the residue was partitioned between ethyl acetate and water and the organic layer was dried over anhydrous MgSO_4_, filtered, and evaporated under reduced pressure. The resulting solid was filtered and washed with methanol and water to give the benzothiazole **2** (508 mg, 31%): ^1^H NMR (400 MHz, CDCl_3_) *δ* 7.95 (s, 1 H, 4′′-H), 7.91 (d, 2 H, *J* = 8.4 Hz, 3′-H, 5′-H), 7.70 (d, 1 H, *J* = 8.4 Hz, 7′′-H), 7.26 (d, 1 H, *J* = 8.4 Hz, 6′′-H), 6.88 (d, 2 H, *J* = 8.8 Hz, 2′-H, 6′-H), 4.22 (q, 2 H, *J* = 7.2 Hz, C*H_2_*CH_3_), 1.64 (s, 6 H, 2×CH_3_), 1.21 (t, 3 H, *J* = 7.2 Hz, CH_2_C*H_3_*); ^13^C NMR (100 MHz, CDCl_3_) *δ* 174.0, 169.6, 158.6, 155.3, 133.4, 132.4, 129.0, 127.0, 125.5, 122.9, 122.4, 118.7, 79.6, 61.9, 25.6, 14.3; LRMS (ESI) *m/z* 376.1 (M+H)^+^.

1 N NaOH (2.0 mL, 2.0 mmol) was added to a stirred solution of compound **2** (508 mg, 1.35 mmol) in 1,4-dioxane (4 mL) and stirred at room temperature for 17 h. The reaction mixture was then partitioned between methylene chloride and water and the aqueous layer was acidified with 6 N HCl to pH 2. The precipitate generated was then filtered and washed with water to give MHY908 (372 mg, 79%) as a white solid: melting point, 190.8–192.0°C; ^1^H NMR (400 MHz, DMSO-*d_6_*) *δ* 8.03 (d, 1 H, *J* = 8.4 Hz, 7′′-H), 7.98 (d, 1 H, *J* = 2.0 Hz, 4′′-H), 7.92 (d, 2 H, *J* = 8.8 Hz, 2′-H, 6′-H), 7.37 (dd, 1 H, *J* = 2.0, 8.4 Hz, 6′′-H), 6.92 (d, 2 H, *J* = 8.8 Hz, 3′-H, 5′-H), 1.55 (s, 6 H, 2× CH_3_); ^13^C NMR (100 MHz, DMSO-*d_6_*) *δ* 175.2, 169.8, 159.0, 155.2, 133.7, 131.9, 129.4, 126.3, 125.8, 124.2, 122.5, 118.9, 79.5, 25.8; LRMS (ESI) *m/z* 348.1 (M+H)^+^; HRMS(ESI) *m/z* 3-(5-{[(thiophen-2-ylmethyl)amino]methyl}furan-2-yl)benzoic acid chloride (M+H)^+^ calcd 348.0461, observed 348.0458.

Alternative synthetic method for MHY908: 1 N NaOH (41.2 mL, 41.2 mmol) was added to a stirred solution of compound **1** (8.106 g, 34.3 mmol) in 1,4-dioxane (50 mL) stirred at room temperature for 4 h. After evaporating volatiles, the residue was partitioned between methylene chloride and water and the aqueous layer so obtained was acidified with 12 N HCl to pH 2. The aqueous layer was then extracted with methylene chloride and the organic layer was dried over MgSO_4_, filtered, and evaporated under reduce pressure to give compound **3** (7.14 g, 99.9%): ^1^H NMR (500 MHz, CDCl_3_) *δ* 10.54 (s, 1 H, COOH), 9.83 (s, 1 H, CHO), 7.79 (d, 2 H, *J* = 8.5 Hz, 3′-H, 5′-H), 6.95 (d, 2 H, *J* = 8.5 Hz, 2′-H, 6′-H), 1.69 (s, 6 H, 2× CH_3_); ^13^C NMR (100 MHz, CDCl_3_) *δ* 191.7, 178.5, 161.0, 132.0, 130.5, 118.5, 79.5, 25.5; LRMS (ESI) *m/z* 209.1 (M+H)^+^. A solution of 2-amino-4-chlorobenzenethiol (60 mg, 0.38 mmol) and compound **3** (78.2 mg, 0.38 mmol) in acetic acid (0.4 mL) was refluxed in the presence of sodium acetate (93.5 mg, 1.13 mmol) for 1 h. After cooling, the reaction mixture was partitioned between ethyl acetate and water and the organic layer was evaporated under reduced pressure. The precipitates generated were filtered and washed with methylene chloride to give MHY908 (46.6 mg, 40%).

### Chromatin immunoprecipitation (ChIP) assays

Chip assays were performed using an EZ ChIP Chromatin immunoprecipitation Kit (Millipore, USA), according to the manufacturer's instructions. Immunoprecipitation was performed using an antibody directed against PPAR α (H-98; Santa Cruz Biotechnology), or PPAR γ (Abcam, USA), or using rabbit IgG (provided in the Millipore kit) as a negative control. After immunoprecipitation, associated DNA was amplified using a primer pair containing PPARs binding site, PPAR response elements (PPREs), PAPR α (forward 5′-ACAGGGTTAGCGGTTGTCAC-3′; reverse 5′-AGAGCAAAGTCTGGGGTGTC-3′), and PPAR γ (forward 5′-GAGCAAGGTCTTCATCATTACG-3′; reverse 5′-CCCCTGGAGCTGGAGTTAC-3′) promoters.

### Transfection and the luciferase assay

For luciferase assays, 0.1 μg of plasmid was transfected into 5×10^4^ cells per well seeded in 24-well plate in 500 μL of DMEM supplemented with 5% FBS, at 37°C in a humidified 95% air/5% CO_2_ atmosphere. Cells were then transfected with lipofectamine transfection reagent and the plasmids used for transfection contained the PPRE-tk-luciferase reporter vector with PPAR α or γ expression vector. After 12 h of transfection, cells were washed, and treated with MHY908, fenofibrate, or rosiglitazone for 6 h. Luciferase activities were then detected using the One-Glo luciferase assay system (Promega, USA), and measured using a TECAN GENios luminescence plate reader (TECAN Instruments, Austria).

### Docking simulations of PPARs and target compounds

The crystal structures of PPAR α and γ were extracted from the PDB archives (entry code PPAR α: 1K7L, PPAR γ: 3DZY) [17], [18] and used as targets. For docking simulations, we used AutoDock4.2 and the tool's manual. Among the many tools available for *in silico* protein-ligand docking, AutoDock4.2 is most commonly used because of its automated docking feature [19]. To define the docking pockets of PPAR α and γ, we used a set of predefined active sites in human PPAR α and γ. Docking simulations were performed between PPAR α or γ and MHY908, fenofibrate, or rosiglitazone (fenofibrate and rosiglitazone were used as reference PPAR α and γ inhibitors, respectively). To prepare compounds for docking simulation, we performed the following steps: [1] 2D structures were converted into 3D structures, [2] charges were calculated, and [3] hydrogen atoms were added using the ChemOffice program (http://www.cambridgesoft.com).

### Shared pharmacophores of target compounds

A pharmacophore is an ensemble of ligand features required for interaction with a specific receptor. A pharmacophore model was generated using the LigandScout 3.0 program [20]. Based on atom types, the chemical features of PPAR α and γ were defined in terms of pharmacophore elements, such as, hydrogen bond acceptors, hydrogen bond donors, positive ionizable areas, negative ionizable areas, hydrophobic interactions, and aromatic rings.

### Animal experimental procedures

Male, 8-week-old, C57BLKS/J-lean and C57BLKS/J-db/db mice were purchased from Japan SLC. Mice were maintained under a 12 h light/dark cycle at 23±1°C and 50±5% RH under specific pathogen-free conditions. db/db mice were allocated to four groups of 8 mice. Animals in the db/db control group were maintained on a normal diet (Feedlab, South Korea). Feeding levels in the calorie restriction (CR) group were calculated by feeding animals with 60% of the individual mean food intake in (db/db control group). Animals in the two MHY908 groups were administered MHY908 mixed in food (based on food intakes, the daily dose of MHY908 was 1 or 3 mg/kg of body weight). Animals were euthanized after four weeks and livers were collected for Western blot analysis. The animal protocol used in this study was reviewed and approved beforehand by the Pusan National University-Institutional Animal Care and Use Committee (PNU-IACUC) with respect to the ethical of procedures and scientific care (Approval Number PNU-2012-0088).

### Biochemical analysis

Blood samples were collected after sacrifice of animals from each group. Kits were used to measure the concentrations of metabolites serum metabolites/cytokine such as insulin (Shibayagi, Japan), glucose and triglyceride (Shinyang, South Korea), adiponectin (Circulex, Japan), and leptin (Morinaga Institute of Biological Science, Japan).

### Western blot analysis

Homogenized samples of liver were boiled for 5 min in gel-loading buffer [0.125 M Tris-HCl, pH 6.8, 4% SDS, 10% 2-mercaptoethanol and 0.2% bromophenol blue] at a volume ratio of 1∶1. Total protein equivalents in samples were separated by sodium dodecyl sulfate-polyacrylamide gel electrophoresis (SDS-PAGE), using 10% acrylamide gels, and then transferred to PVDF membranes at 80 V and 1.5 h, using a semi-dry transfer system. Membranes were immediately placed in blocking buffer (10 mmol Tris (pH 7.5), 100 mmol NaCl, 0.1% Tween 20, and 5% non-fat milk). Blots were allowed to block at room temperature for 30 min, and then membranes were incubated with specific primary antibody (PPAR α or PPAR γ, Santa Cruz) at 4°C overnight, followed by horse radish peroxidase-conjugated anti-rabbit antibody (Santa Cruz, USA) at room temperature for 1.5 h. Antibody labeling was detected using a Westsave^TM^ (Abfrontier, South Korea). Pre-stained protein markers were used for molecular weight determinations.

### Statistical analysis

All results are expressed as means ± SEMs. Treatments were compared by one-way ANOVA followed by Dunnett's test. Statistical significance was accepted for *P* values <0.05.

## Results

### MHY908 increased PPAR α and γ binding affinities for PPRE and their transcriptional activities

**Figure 2:**
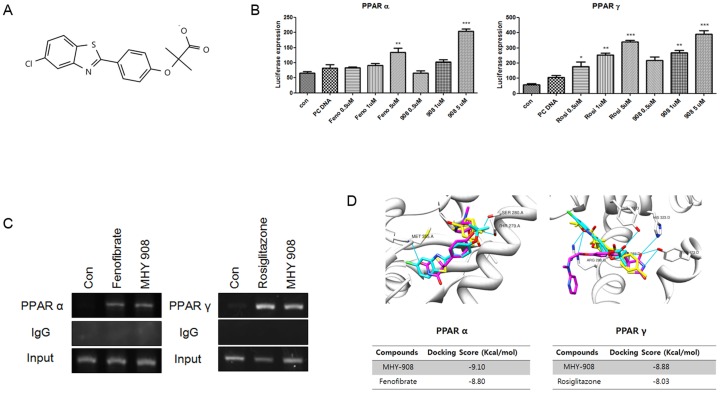
MHY908 functioned as a PPAR α/γ dual agonist. (A) The chemical structure of MHY908. (B) ChIP assay for PPARs binding to PPRE of the PPAR α or γ promoter. The extracted genomic DNA was subjected to immunoprecipitation using an antibody against PPAR α and PPAR γ or IgG as a negative control. Amplification derived from unprecipitated chromatin is shown (input). (C) MHY908 induced the transcriptional activities of PPAR α and γ, respectively. One-factor ANOVA was used to determine the significances of differences: *** p<0.001, ** p<0.01 and * p<0.05 versus the PC DNA. (D) The docking simulation between PPAR α or γ and their activators or MHY908. The Grey zone indicates the active site. Magenta indicates MHY908, and cyan indicates fenofibrate or rosiglitazone (positive controls). The binding energies of compounds for PPAR α were −9.10 kcal/mol (MHY908) and −8.80 kcal/mol (fenofibrate) or and for PPAR γ were −8.88 kcal/mol (MHY908) and −8.03 kcal/mol (rosiglitazone).

A chromatin immunoprecipitation (ChIP) assay was used to determine whether PPAR α and γ activation by MHY908 (Fig. 2A) results in PPAR α and PPAR γ binding to PPRE. We observed that interactions between PPAR α or PPAR γ and PPRE were induced by MHY908 (Fig. 2B), indicating that MHY908 is a novel agonist of PPAR α and PPAR γ. We also tested the activating effects of fenofibrate, rosiglitazone, and MHY908 on PPAR α and PPAR γ, using a luciferase reporter gene assay. As shown in Fig. 2C, MHY908 induced higher PPAR α and PPAR γ dependent reporter activities in AC2F rat liver cells than fenofibrate and rosiglitazone, respectively. The results suggest that MHY908 is a potent dual agonist of PPAR α and PPAR γ.

### MHY908 interacted with the Ligand Binding Domain (LBD) of PPAR α and PPAR γ

The docking simulation exercise produced significant scores. The binding energies of MHY908 and PPAR α or PPAR γ were −9.10 and −8.88 kcal/mol, respectively, and the binding energies of fenofibrate and rosiglitazone for PPAR α and PPAR γ were −8.80 and −8.03 kcal/mol, respectively. The docking score between a ligand and receptor is arrived at by summing energy terms, such as, electrostatic energy, van der Waals energy, and solvation energy, and in the present study docking simulation showed that the binding affinities of MHY908 were greater than those of fenofibrate and rosiglitazone (Fig. 2D). In addition, we searched for hydrogen binding interactions between PPAR α or γ and MHY908, fenofibrate, or rosiglitazone. The Ser-280 residue of PPAR α was found to be mainly responsible for hydrogen bonding with MHY908. However, the Ala-333 and Thr-279 residues of PPAR α were predicted to be involved in hydrogen bonding with fenofibrate (Fig. 2D), and the Tyr-327 residue of PPAR γ was predicted to be involved in hydrogen bonding with MHY908. However, rosiglitazone was not found to have a hydrogen bonding interaction with PPAR γ. Accordingly, these residues might function as key determinants of inhibitor activity and total binding affinity.

### MHY908 improved basal serum lipid and glucose metabolisms in db/db mice

**Figure 3:**
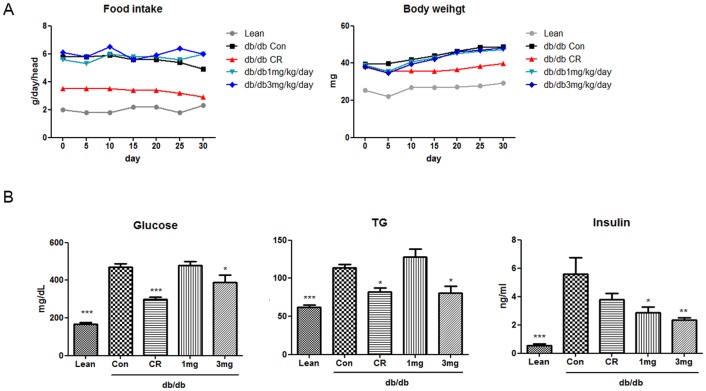
Effect of MHY908 on body weight, plasma glucose, TG, and insulin levels in db/db mice. Mice were treated for 4/kg/day in food. Food intakes and body weights were similar in the Con group and MHY908- treated groups (n = 8/group, eight-weeks old). (A) Changes in body weights. (B) A series of plasma profiles from CR and MHY908 treated db/db mice. One-factor ANOVA was used to determine the significances of differences: *** p<0.001, ** p<0.01 and * p<0.05 versus the Con group. Lean; db/m mice, Con; db/db mice, CR; calorie restriction, 1 mg; MHY 908 1 mg/kg/day, 3 mg; MHY 908 3 mg/kg/day.

We also investigated the pharmacological effects of MHY908 in db/db mice. Serum glucose, TG, and insulin profiles were analyzed to determine whether MHY908 attenuates metabolic abnormalities in obese and diabetic subjects. In db/db mice, MHY908 significantly reduced serum glucose, TG, and insulin levels (Fig. 3B). Furthermore, these effects of MHY908 on serum profiles were similar to those of CR. These data suggest that MHY908 could efficiently redress serum glucose, insulin, and lipid abnormalities without significant body weight gain (Fig. 3A).

### MHY908 reduced fat in liver in db/db mice

**Figure 4:**
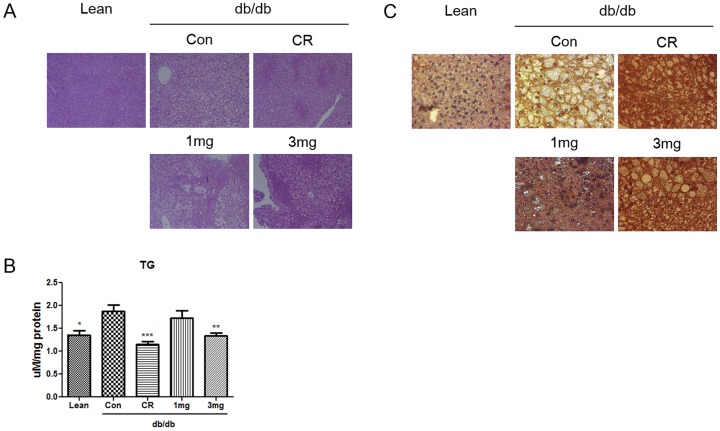
MHY908 improved hepatic steatosis in db/db mouse livers. (A) A histological examination based on hematoxylin-eosin staining showed marked fatty changes in the livers of db/db mice. (Original magnification 100 X) (B) Accumulated lipids in the livers of MHY908-treated db/db mice. One-factor ANOVA was used to determine the significances of differences: *** p<0.001, ** p<0.01 and * p<0.05 versus the Con group. (C) CPT-1 expression was assessed by immunohistochemistry (Original magnification 200 X). Lean; db/m mice, Con; db/db mice, CR; calorie restriction, 1 mg; MHY 908 1 mg/kg/day, 3 mg; MHY 908 3 mg/kg/day.

Fatty liver is a complication of type 2 diabetes. Since MHY908 was developed and identified as a PPAR α/γ dual agonist, we examined whether MHY908 attenuates fatty liver in db/db mice. Histological analysis showed that lipid droplets accumulated in the livers of db/db mice, but that 3 mg/kg of MHY908 in food daily for 4 weeks markedly reduced this accumulation (Fig. 4A). To confirm this effect, we also examined hepatic TG contents. As shown in Fig. 4B, hepatic TG contents were high in db/db controls, but that like observed in CR mice, MHY908 greatly decreased hepatic TG levels (Fig. 4B). As was expected, MHY908 increased the expression of CPT-1 (the fatty acid oxidation gene) in liver (Fig. 4C). These results strongly suggest that MHY908 reduces fat levels in liver.

### MHY908 attenuated ER stress-induced insulin resistance in the livers of db/db mice

**Figure 5:**
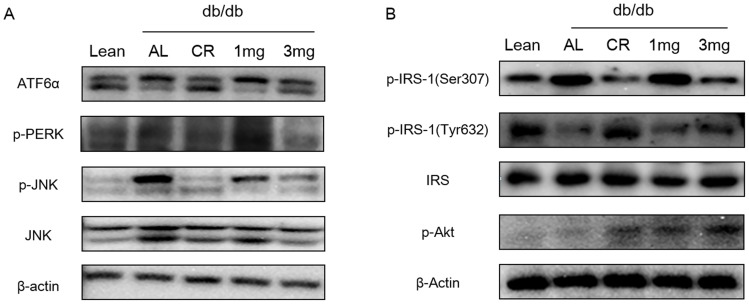
MHY908 attenuated ER stress induced insulin resistance in the livers of db/db mice. (A) Results of Western-blot analysis of the livers of db/db mice; the ER stress markers p-IRE, p-PERK, and p-JNK are shown. CR and MHY908 decreased ER stress marker protein levels in db/db livers, and (B) CR and MHY908 improved insulin signaling in db/db livers. Lean; db/m mice, Con; db/db mice, CR; calorie restriction, 1 mg; MHY 908 1 mg/kg/day, 3 mg; MHY 908 3 mg/kg/day.

It has been recently suggested that ER stress plays a central role in the development of insulin resistance and diabetes via the JNK-mediated inhibition of insulin activity [3]. To characterize the molecular mechanisms underlying the anti-diabetic effects of MHY908, we examined whether MHY908 alleviates ER stress in the livers of db/db mice. As shown in Fig. 5A, markers of ER stress, such as, IRE and PERK, were higher in db/db mice than in control mice, but CR or MHY908 at 3 mg/kg/day reduced ER stress response in the liver of db/db mice. Consistent with these results, JNK phosphorylation was also significantly suppressed in liver by CR and by treatment with MHY908 at 3 mg/kg. In addition, MHY908 also suppressed the serine phosphorylation of IRS-1, and thus, improved tyrosine phosphorylation in IRS-1 and threonine phosphorylation in Akt (Fig. 5B).

### MHY908 reduced serum leptin and adiponectin levels in db/db mice

**Figure 6:**
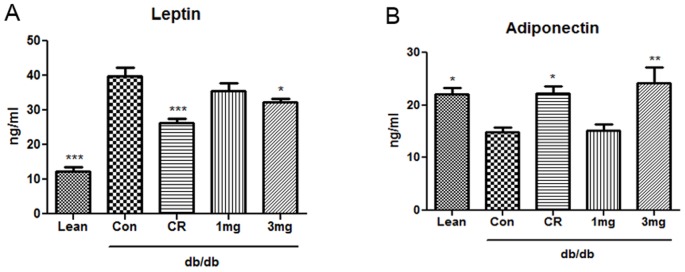
MHY908 modulated serum leptin and adiponectin levels in db/db mice. (A) Serum concentrations of leptin (n = 6) (B) Serum concentrations of adiponectin (n = 6) One-factor ANOVA was used to determine the significances of differences: *** p<0.001, ** p<0.01 and * p<0.05 versus the Con group. Lean; db/m mice, Con; db/db mice, CR; calorie restriction, 1 mg; MHY 908 1 mg/kg/day, 3 mg; MHY 908 3 mg/kg/day.

An elevated circulating leptin level is a marker of leptin resistance and is independently associated with insulin resistance, and is commonly observed in the obese. Therefore, we examined whether MHY908 down-regulates serum leptin levels in db/db mice (Fig. 6A). Because diabetic db/db mice have dysfunctional leptin receptors, serum leptin levels were significantly higher in the Con group than db/m group, whereas the CR or MHY908 (3 mg/kg) groups had significantly lower serum leptin levels than the Con group (Fig. 6B). Recently, elevated plasma adiponectin concentrations have reported to affect insulin sensitivity [21], [22]. Fig. 6B shows that serum adiponectin levels were elevated in the CR and MHY908 3 mg/kg groups. These results suggest that MHY908 improves insulin resistance by modulating adipokine.

## Discussion

Type 2 diabetes and its related complications are serious worldwide health problems, and thus, an aggressive approach that modulates diabetes using anti-diabetic agents that ameliorate insulin resistance and hyperlipidemia is needed. The therapeutic advantages of PPAR α and/or PPAR γ agonists for the treatment of metabolic abnormalities prompted the development of PPAR α/γ dual agonists, such as, razaglitazar, tesaglitazar, and muraglitazar, [23], [24], [25] which specifically activate PPAR α and PPAR γ. Unfortunately, clinical trials on a PPAR α/γ dual agonists, such as, tesaglitazar and muraglitazar, were discontinued due to their undesirable side effects. Muraglitazar was associated with an increased incidence of heart failure [26] and tesaglitazar was associated with decreased glomerular filtration. In the current study, we designed and synthesized several non-glitazar based compounds. Initially, we designed and synthesized 2-(substituted phenyl)benzothiazole analogs as tyrosinase inhibitors and later, found that the compounds increased PPAR α and γ activities. In order to study structure-activity relationship, compounds with a chloro or trifluoromethyl group on the benzothiazole ring were synthesized and evaluated for PPAR α and γ activation. These compounds also showed good PPAR α and γ activation. Fatty acids have a “carboxylic acid” functionality and are known to be natural PPAR ligands. Also, fenofibrate and clofibrate, well-known. PPAR α activators have a “carboxylic acid” functionality (exactly “isobutyric acid”). Therefore, for enhancing PPAR α activity, introduction of “isobutyric acid” into 2-(substituted phenyl)benzothiazole analogs was tried (Fig. 1). MHY 908 exhibited higher PPAR α/γ activity on TR-FRET PPARs competitive binding assay and lower cytotoxicity on MTT assay than the other compounds. And then, we evaluated the characteristics and protective effects of MHY908 against diabetes in *in vitro* and *in vivo*.

MHY908 showed agonistic activities for PPAR α and γ. Interestingly, MHY908 more potently activated PPAR α and PPAR γ than fenofibrate and rosiglitazone, respectively (Fig. 2C). The high potency of MHY908 with respect to the activations of PPAR α and γ can be explained by our docking simulation results. We found that fenofibrate (binding energy: −8.80 kcal/mol) does not interact with any acidic amino acid in PPAR α, but that MHY908 (binding energy: −9.10 kcal/mol) interacts with two acidic amino acids (Asp-353 and Glu-356) within 5 Å of PPAR α isoforms (Fig. 2D and Table 1). Similarly, rosiglitazone (binding energy: −8.03 kcal/mol) was not found to interact with any acidic amino acids in PPAR γ, but MHY908 (binding energy: −8.88 kcal/mol) interacts with the acidic amino acid Glu-295 within 5 Å of the PPAR γ isoforms (Fig. 2D and Table 1). Furthermore, the negatively charged 2-methylpropionic acid group of MHY908 was found to form three strong hydrogen bonds with PPAR α and with PPAR γ (Ser-280 and Met-355 for PPAR α and Tyr-327 for PPAR γ). It is well-known that the LBD of nuclear hormone receptors undergo conformational changes when they interact with their ligands, and that these changes activate nuclear receptors [27]. The stronger binding between MHY908 and both PPAR isoforms than between the two positive controls and the isoforms suggests that MHY908 is a better agonist.

**Table 1:** Detailed information on the hydrogen bonding networks formed by fenofibrate, rosiglitazone, and MHY908.

Receptor	Compound	Phosphorylation Position	Å<5 acidic amino acid
PPAR α	Fenofibrate	Thr-279, Ser-280	None
	Rosiglitazone	None	None
	MHY 908	Ser-280, Met-355	Asp-353, Glu-356
PPAR γ	Fenofibrate	Arg-288	None
	Rosiglitazone	Ser-289, His-323, Tyr-473	None
	MHY 908	Tyr-327	Glu-295

Many authors have concluded that PPAR α/γ dual agonists have been identified and tested obese and insulin resistant individuals [12], [13], [25] and CR improves insulin sensitivity in db/db mice, and thus, we compared CR with MHY908. When administered to db/db mice, CR with 3 mg/kg of MHY908 significantly ameliorated insulin resistance and improved fatty acid and glucose metabolism in db/db mice (Fig 3). These observations suggest that, like CR mimetics, MHY908 could provide a suitable therapeutic approach to the treatment of type 2 diabetes, metabolic syndrome, and insulin resistance.

Fatty liver is a common complication in obese [28] and in type 2 diabetic patients and is closely associated with insulin resistance [29]. Furthermore, the strong association between hepatic steatosis and insulin resistance in humans [30] and animal models [31], [32] suggests that insulin resistance is involved in the pathogenesis of obesity-related fatty liver disease. In this study, we found that and treatment with 3 mg/kg of MHY908 prevented hepatic steatosis by reducing hepatic TG levels in db/db mice (Fig. 4B). In fact, MHY908 increased the expression of CPT-1 (the PPAR α target gene) in livers of db/db mice (Fig. 4C), suggesting that MHY908 promotes hepatic lipid oxidation. Furthermore, the beneficial effects of several PPAR α/γ dual agonists have also been attributed to the modulation in fatty liver [25], [33].

As ER is a central organelle involved in lipid synthesis, protein folding, and protein maturation, ER stress is receiving greater research attention as a cause of insulin resistance via JNK activation, and thus, the inhibition of insulin signaling [3], [34]. Therefore, we investigated whether MHY908 could alleviate ER stress and improve insulin signaling in the livers of db/db mice. Markers of ER stress and JNK phosphorylation were elevated in db/db mice, but MHY908 significantly reduced these ER stress markers and JNK phosphorylation (Fig. 5A). Consistent with these results, MHY908 down-regulated IRS-1 serine phosphorylation and enhanced both IRS-1 tyrosine phosphorylation and Akt threonine phosphorylation (Fig 5B). Our results are in agreement with those of a previous study, in which treatment of db/db mice with a PPAR dual agonist reduced ER stress and improved insulin signaling in the livers of db/db mice [35].

Leptin levels reflect the amount of stored fat and degree of energy imbalance. A prolonged over-nutritional state greatly increases fat stores [36] by insulin stimulation. In our previous study, it was shown that troglitazone reduces leptin expression in adipocytes [37], and in the present study, we found that MHY908 (and CR) down-regulated leptin levels (Fig. 6A). Adiponectin, a specific PPAR γ-induced adipokine has recently been shown to enhance lipid metabolism and insulin action in liver [38], and like other PPAR γ agonists, such as, rosiglitazone and ragaglitazar, MHY908 dramatically increased adiponectin in serum after levels, in fact, more than CR (Fig. 6B). Furthermore, our results show that leptin and adiponectin levels were reduced, when obese mice are treated with MHY908. It has been reported that adiponectin and leptin levels, which are both modulated by PPAR α/γ dual agonists, are also influenced by type 2 diabetes [39], [40]. Accordingly, these reports support the notion that MHY908 has potential use as a treatment for metabolic syndrome and insulin resistance associated with in type 2 diabetes.

**Figure 7:**
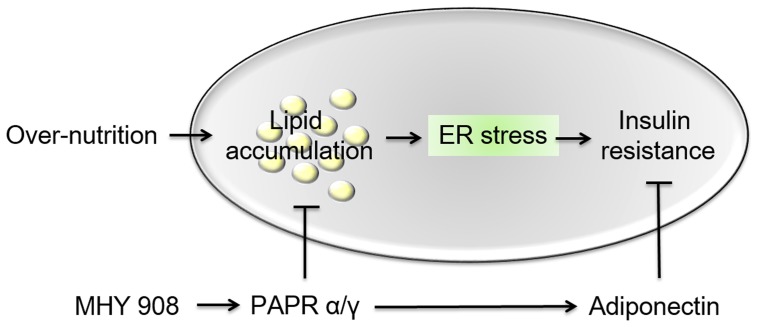
Possible mechanism of the effects of MHY908 on overnutrition-induced insulin resistance. We suggest that overnutrition induced lipid accumulation and led to insulin resistance by increasing ER stress, and that MHY908 downregulated ER stress and improved insulin signaling in the livers of db/db mice.

In summary, MHY908 exhibited greater PPAR α and γ agonistic activities than fenofibrate and rosiglitazone, which are known PPAR α and γ agonists, respectively. In addition, MHY908 regulated glucose and lipid metabolisms, and ameliorated ER stress and insulin signaling in diabetic animal model. Furthermore, MHY908 also modulated serum leptin and adiponectin levels. Therefore, we concluded that MHY908 has the potential to prevent the up-regulation of ER stress and insulin resistance resulting from overnutrition-induced lipid accumulation by activating PPAR α and γ (Fig. 7). In addition, its effects on ER stress and insulin resistance support the candidature of MHY908 as a drug for the treatment of type 2 diabetes.
